# Deep Learning-Enhanced T1-Weighted Imaging for Breast MRI at 1.5T

**DOI:** 10.3390/diagnostics15131681

**Published:** 2025-07-01

**Authors:** Susann-Cathrin Olthof, Marcel Dominik Nickel, Elisabeth Weiland, Daniel Leyhr, Saif Afat, Konstantin Nikolaou, Heike Preibsch

**Affiliations:** 1 Department of Diagnostic and Interventional Radiology, University Hospital of Tuebingen, 72076 Tuebingen, Germany; 2 Research & Clinical Translation, Magnetic Resonance, Siemens Healthineers AG, 91052 Erlangen, Germany; 3 Faculty of Economics and Social Sciences, Institute of Sports Science & Methods Center, University of Tuebingen, 72074 Tuebingen, Germany; 4 Cluster of Excellence iFIT (EXC 2180) “Image Guided and Functionally Instructed Tumor Therapies”, University of Tuebingen, 72076 Tuebingen, Germany

**Keywords:** MRI, breast cancer, deep learning, diagnostic imaging

## Abstract

**Background/Objectives**: Assessment of a novel deep-learning (DL)-based T1w volumetric interpolated breath-hold (VIBE_DL_) sequence in breast MRI in comparison with standard VIBE (VIBE_Std_) for image quality evaluation. **Methods**: Prospective study of 52 breast cancer patients examined at 1.5T breast MRI with T1w VIBE_Std_ and T1 VIBE_DL_ sequence. T1w VIBE_DL_ was integrated as an additional early non-contrast and a delayed post-contrast scan. Two radiologists independently scored T1w VIBE _Std/DL_ sequences both pre- and post-contrast and their calculated subtractions (SUBs) for image quality, sharpness, (motion)–artifacts, perceived signal-to-noise and diagnostic confidence with a Likert-scale from 1: Non-diagnostic to 5: Excellent. Lesion diameter was evaluated on the SUB for T1w VIBE_Std_/_DL_. All lesions were visually evaluated in T1w VIBE_Std_/_DL_ pre- and post-contrast and their subtractions. Statistics included correlation analyses and paired t-tests. **Results**: Significantly higher Likert scale values were detected in the pre-contrast T1w VIBE_DL_ compared to the T1w VIBE_Std_ for image quality (each *p* < 0.001), image sharpness (*p* < 0.001), SNR (*p* < 0.001), and diagnostic confidence (*p* < 0.010). Significantly higher values for image quality (*p* < 0.001 in each case), image sharpness (*p* < 0.001), SNR (*p* < 0.001), and artifacts (*p* < 0.001) were detected in the post-contrast T1w VIBE_DL_ and in the SUB. SUB_DL_ provided superior diagnostic certainty compared to SUB_Std_ in one reader (*p* = 0.083 or *p* = 0.004). **Conclusions**: Deep learning-enhanced T1w VIBE_DL_ at 1.5T breast MRI offers superior image quality compared to T1w VIBE_Std_.

## 1. Introduction

Dynamic contrast-enhanced (DCE) breast MRI is the imaging modality with the highest sensitivity for the detection of breast cancer [1]. Therefore, breast MRI plays a crucial role in breast cancer screening in high-risk women with genetic mutations like breast cancer gene (BRCA) 1 or 2 [2], as well as for the local staging in women with biopsy-proven breast cancer [3]. Patients with dense breast tissue, invasive lobular carcinoma, suspected multicentric tumor extent, and potential infiltration of the chest wall benefit from preoperative breast MR [4]. Monitoring the response to neoadjuvant chemotherapy is another key application of breast MRI [5]. 

The dynamic T1w sequence pre- and post-contrast media application is essential for breast MRI. Subtraction images are calculated, which are mandatory if T1w sequences are acquired without fat suppression [5].

Over the last 30 years, technological development has enabled the application of machine learning in imaging, among other things, to achieve more precise diagnostics, particularly in tumor detection and classification [6,7]. DL as a subfield of machine learning is preferred, as it offers the findings faster and more accurately, as it uses multiple neural networks comparable to human cerebral neurons [6,7,8,9].

Deep learning methods are divided into supervised, unsupervised, and semi-supervised learning models for disease detection and diagnosis [10]. Regarding the supervised models, so far, DL has enabled the detection of asymmetry and skin thickness based on Dynamic Time Warping (DTW) and Growing Region Seed (GRS) for feature extraction in 114 mammograms, including 25 BI-RADS 4^®^ or 5 lesions [11].

The DL application in bilateral tomosynthesis for the detection of parenchymal asymmetry in 115 patients with histologically proven one-sided breast carcinoma was superior to the unilateral trained model [12].

In ultrasound, DL enabled the differentiation of malignant and benign breast lesions by applying a stacked denoising auto-encoder (SDAE) on the computer-aided diagnosis (CAD) in 520 breast examinations, resulting in 245 histologically proven carcinomas [13]. 

In MRI, DL was able to reduce the biopsy rate by 20% of BI-RADS 4 findings in a study of over 21,000 breast MRIs of over 13,000 patients [14]. 

Within image evaluation, the DL technique is also applied for image reconstruction in CT and MRI, as well as the post-processing steps, including image enhancement and artefact correction [13,15]. Novel DL reconstruction algorithms have been investigated for faster acquisition time, while significantly improving the image quality for T1w VIBE in the abdomen at 1.5T and 3T and for T2w imaging of the prostate, as well as for diffusion-weighted imaging of the breast (DWI) [16,17,18,19]. Moreover, DL-enhanced sequences are used over a broad range of applications, even down to a field strength of 0.05T, where they enable fast 3D brain MRI [20].

The goal of this study was to compare the image quality of a new T1w VIBE with a DL reconstruction algorithm with conventional VIBE in clinical routine in patients with histologically proven breast cancer undergoing breast MRI.

## 2. Materials and Methods

### 2.1. Patient Cohort

This unicenter, prospective study was conducted in accordance with the Declaration of Helsinki, and the protocol was approved by the Ethics Committee of our hospital (055/201BO2). All patients with histologically proven breast cancer, examined via breast MRI for clinically indicated, primary staging at our institution, were asked to participate in this prospective study from May 2023 to February 2024. Comparable to other feasibility studies, which analyzed whether DL VIBE provides the required image quality for clinical application, our small study group consisted of 60 patients [16,18,21]. A post hoc sensitivity analysis was conducted in order to detect the minimum detectable population effect size for differences between VIBE_Std_ and VIBE_DL_ using G*Power (version 3.1.9.7; α = 0.05, 1 − β = 0.80, two-tailed). The analyses revealed sufficient sensitivity to detect small-to-moderate differences (d = 0.40).

**Figure 1:**
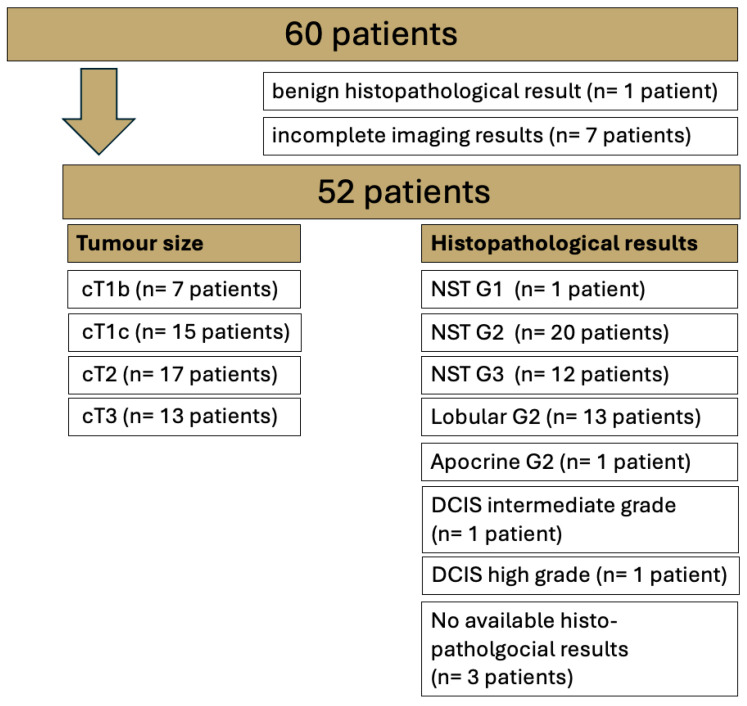
Overview of the entire study cohort examined via 1.5T breast MRI with T1 VIBE Standard (Std) and T1 VIBE deep learning (DL). No special type (NST); ductal carcinoma in situ (DCIS).

The study protocol consisted of a T1w VIBE_DL_ sequence pre- and post-contrast media application in addition to the conventional protocol of T2w TIRM and T1w VIBE Std and DL sequences pre- and post-contrast media application. For details on the histological breast cancer subtypes and the tumor size of the patients included in this study, see Figure 1.

### 2.2. Image Acquisition

All images were acquired with a 1.5T MRI system (MAGNETOM Aera, Siemens Healthineers, Forchheim, Germany). Patients were examined in the prone position with a dedicated seven-channel bilateral breast coil (Siemens Healthineers, Erlangen, Germany) and received a body weight-adapted dose of i.v. Gadovist (Bayer Healthcare, Berlin, Germany; 0.1 mmol gadobutrol/kg body weight). Clinical breast imaging was performed with a T2w fat-suppressed turbo inversion recovery magnitude sequence (T2w TIRM) and non-fat suppressed 3D T1w before and after contrast agent application (T1w VIBE_Std_ TE 4.77 ms, TR 7.73 ms, TA 73s, phase and slice partial Fourier 7/8 and 5/8; T1 VIBE_DL_ TE 4.77 ms, TR 7.69 ms, ETA 73s, phase and slice partial Fourier 6/8 (Table 1)). In addition, a VIBE_DL_ sequence was integrated into the same clinical examination as an early pre-contrast scan and a delayed post-contrast phase to prevent any interference with our clinical imaging standards. The applied DL T1w VIBE technique is a research application package, which was provided by Siemens Healthineers.

### 2.3. Deep Learning-Accelerated VIBE_DL_ Sequence

Using the inherent image enhancement of a deep learning-based reconstruction, higher accelerated sampling and consequently higher resolution can be achieved by the VIBE_DL_ sequence in the same acquisition time. The reconstruction employs two sequential stages that were developed independently.

**Figure 2:**
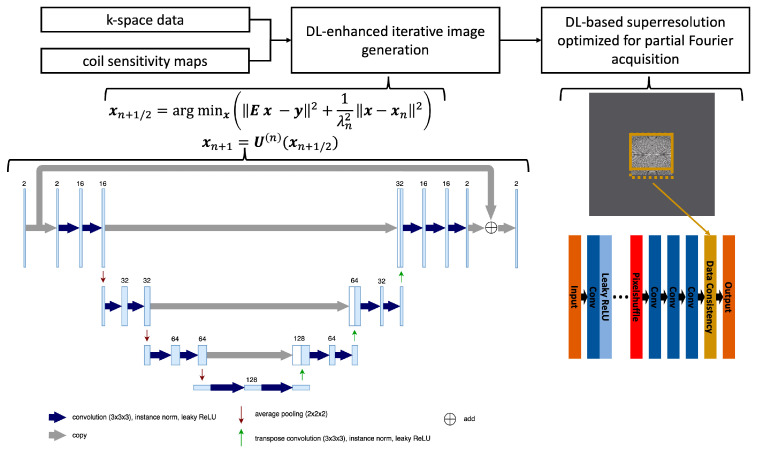
Flowchart of the reconstruction process using undersampled k-space data as well as precalculated coil sensitivity maps as input, performing the image generation through six iterations that alternate between a parallel imaging model and a network-based image enhancement, and finally interpolating the image using a deep learning-based super-resolution tailored to the selected partial Fourier sampling in the acquisition. Furthermore, the conventional 3D U-net architecture used in each iteration is detailed with the employed hyperparameters.

In the first stage, images are generated from k-space data on the acquired resolution using a network architecture inspired by unrolled variational networks [22]. The algorithm receives precalculated coil sensitivity maps as well as undersampled k-space data as input and performs six iterations that alternate between parallel imaging-based data consistency and neural network-based image enhancement. Using about 500 fully sampled datasets obtained from healthy volunteers on 1.5T and 3T scanners (MAGNETOM scanners, Siemens Healthineers, Forchheim, Germany) in the head, abdomen, and pelvis, the network was trained end-to-end in a supervised manner with about 5000 derived sub-volumes. Conventional 3D U-nets were chosen as image enhancement networks, the L1-norm was chosen as the loss function, and Adam was chosen as the optimizer. For more details, we refer to Figure 2, which illustrates the network architecture. The obtained model parameters were then integrated into the reconstruction pipeline of the scanner for prospective use. The implementation was previously explored for dynamic liver imaging, with more details given in Ref. [13].

In the second stage, the obtained complex-valued image volumes were non-linearly interpolated by a factor of two in all spatial directions using a deep learning-based super-resolution algorithm. For supervised training using a similar dataset as employed for the first stage, the input data were generated through retrospective downsampling, and partial Fourier sampling, corresponding to the prospective acquisition, was simulated in the Fourier domain. Consequently, the obtained model parameters were tailored to the selected partial Fourier settings. Similar to the k-space reconstruction, these parameters were integrated into the reconstruction pipeline of the scanner, and we refer to Refs. [6,8] for more details.

The overall deep learning-based image reconstruction was provided by Siemens Healthineers as a research application.

### 2.4. Image Analysis

Image analysis was performed on the pre-contrast scans of T1w VIBE_Std_ and T1w VIBE_DL_, the latest available post-contrast scan for both sequences, and corresponding subtractions (SUBs) by two radiologists (H.P. with 13 years and S-C.O. with 7 years of experience in reading breast MRI). Although the latest post-contrast scans for T1w VIBE_Std_ are not the most clinically relevant, these time points were chosen for the analysis, as they provide the closest time match to the delayed post-contrast phase of T1w VIBE_DL_.

For lesion analysis, only biopsy-proven malignant lesions were examined. A total of 49 of the 52 included patients were surgically treated in-house, and histopathological specimens were analyzed at our local histopathology institute (for further details, see Figure 1). Benign lesions (n = 1) were omitted from the analysis.

Each reader evaluated the pre- and post-contrast media images as well as the calculated SUB for T1w VIBE_Std_ and _DL_ sequences qualitatively in our standard postprocessing software (syngo.via, 9.4, Siemens Healthineers, Erlangen, Germany). Qualitative image evaluation was based on a five-point Likert scale (with 1 for non-diagnostic imaging, 2 for poor, 3 for moderate, 4 for good, and 5 for excellent) for motion artefacts, image quality, artefacts, sharpness, signal-to-noise ratio (SNR), and diagnostic confidence. Motion artefacts included those caused by breathing, which cannot be excluded effectively even with craniocaudal fixation of the breast.

Diagnostic confidence of the unsubtracted pre- and post-contrast T1w VIBE_DL_ and _Std_ sequence referred to the visibility of the tumor lesion in those non-diagnostic images for the study purpose of image quality evaluation.

Quantitative analysis included the diameter of the malignant lesion in T1w VIBE _Std_ and T1w VIBE _DL_ compared to our gold standard, the 2nd SUB of T1w VIBE_Std_.

### 2.5. Statistical Analysis

SPSS (version 28, IBM, Chicago, IL, USA) was used for statistical calculations. The determination of the interrater reliability was analyzed based on contingency tables and the resulting percentage of agreement between raters, as the utilized Likert scales were often not fully utilized and, thus, the calculation of correlation values does not provide meaningful results. Outcome differences between VIBE_Std_ and VIBE_DL_ were determined via paired *t*-tests. Results with *p*-values of ≤0.05 were considered statistically significant.

## 3. Results

### 3.1. Patients

Of the 60 patients who gave written informed consent, 1 patient had histologically proven benign disease (adenosis) and another 7 patients had incomplete imaging results (Figure 1). The mean age of the 52 female patients included in this study was 55.7 years (SD 22.1 years).

### 3.2. Qualitative Image Evaluation

#### 3.2.1. Qualitative Image Evaluation for T1w VIBE Pre-Contrast

T1w VIBE_DL_ revealed superior image quality compared to T1w VIBE_Std_ for the image sharpness and SNR, as well as a higher diagnostic confidence in both readers (Table 2; all *p* ≤ 0.05). One reader detected significantly more artefacts in T1w VIBE_DL_ compared to T1w VIBE_Std_, without affecting the diagnostic confidence (*p* < 0.001), however. Motion artefacts were comparable in T1w VIBE_Std_ and the new T1w VIBE_DL_ in both readers (*p* > 0.99 and *p* = 0.322, respectively). The percentage of agreement between raters ranged between 38% for artefacts and 96% for the evaluation of motion artefacts (Table 2).

#### 3.2.2. Qualitative Image Evaluation for T1w VIBE Post-Contrast

T1w VIBE_DL_ offered superior results compared to T1w VIBE_Std_ for image quality, sharpness, and the SNR in both readers (*p* < 0.001, Table 3). Also, the diagnostic confidence was rated superior in T1w VIBE_DL_ compared to T1w VIBE_Std_ in one reader (*p* = 0.001). Regarding the motion artefacts, no significant differences between the two sequences were noted in both readers (*p* = 0.261 and *p* = 0.322, respectively). However, a significantly higher number of image artefacts were detected in T1w VIBE_DL_ compared to the Std sequence in both readers (*p* < 0.001, respectively). The percentage of agreement between raters ranged from 51% for reader 1 for sharpness and up to 92% for reader 1 regarding the motion artefacts.

### 3.3. Qualitative Image Evaluation for Post-Contrast Subtraction Images (SUB)

**Figure 3:**
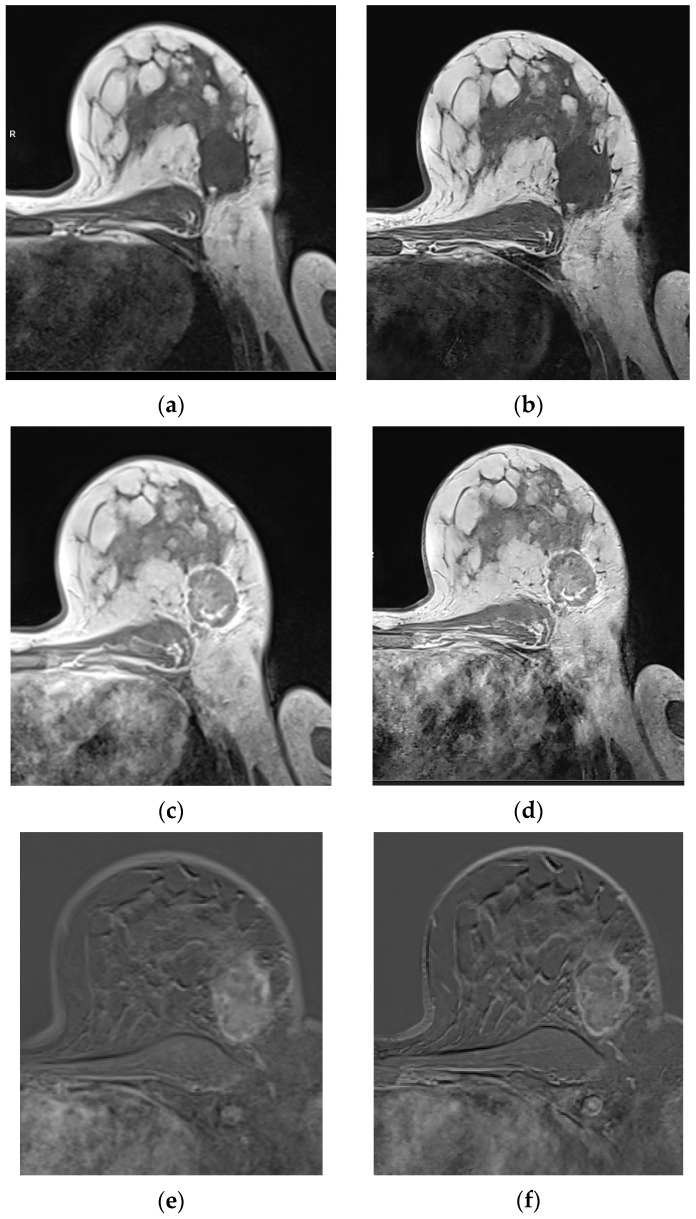
Patient with histologically proven no-special type (NST) breast carcinoma G2 on the left side, with a lesion diameter of 45mm (pT2). Compared to T1 VIBE_Std_ pre-contrast (**a**), post-contrast (**c**), and the last SUB (**e**), the T1 VIBE_DL_ pre-contrast (**b**), post-contrast (**d**), and the SUB (**f**) display the tumor with higher sharpness, without affecting diagnostic confidence. Based on the evaluation of the latest SUB, there are relevant motion artifacts in T1 VIBE SUB_Std_ and _DL_, based on the study design.

**Figure 4:**
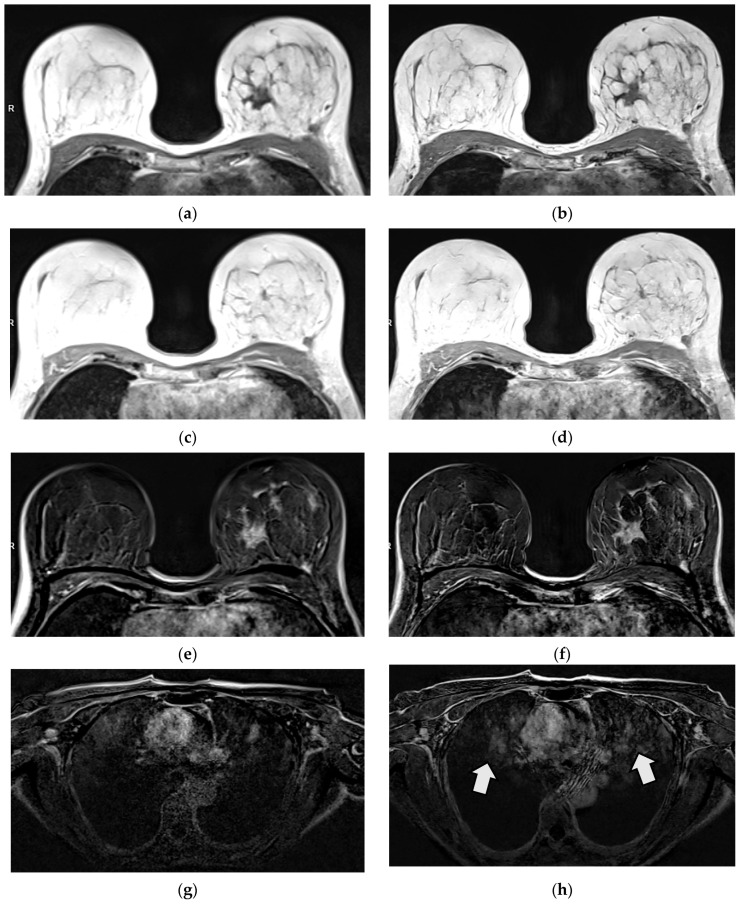
Patient with histologically proven invasive lobular breast carcinoma (ILC) G2 on the left side. Tumor margins are sharper in T1 VIBE_DL_ (**b**) compared to T1 VIBE_Std_ (**a**) in pre-contrast images. No tumor assessment is possible in non-diagnostic post-contrast images in T1 VIBE_Std_ and _DL_ (**c**,**d**). The tumor is displayed sharply in T1 VIBE SUB_DL_ compared to T1 VIBE SUB_Std_ (**e**,**f**), while more artefacts outside the area of interest were detected in T1 VIBE_DL_, e.g., in the mediastinum (**g**,**h**).

T1 VIBE subtraction images of the T1w VIBE_DL_ (SUBs) showed superior results compared to the SUB of T1w VIBE_Std_ for image quality, sharpness, and the SNR in both readers (*p* < 0.001; Table 3, Figure 3). Diagnostic confidence was also rated significantly superior in one reader (*p* = 0.004). Motion artefacts were comparable between T1w VIBE_DL_ and _Std_ in both readers (*p* = 0.103 and 0.766). However, significantly more artifacts in the mediastinum apart from the diagnostic relevant area were detected in the SUBs of T1w VIBE_DL_ compared to Std in both readers (*p* < 0.001; Figure 4). The percentage of agreement between raters ranged from 34% for artefacts in reader 2 and 91% for diagnostic confidence in reader 2 (Table 4).

### 3.4. Quantitative Image Evaluation: Lesion Visibility and Diameter

The primary tumor was visible in 50/52 T1w VIBE_Std_ and T1w VIBE_DL_ pre- and post-contrast scans. In the diagnostic relevant SUB of T1w VIBE_Std_ and _DL_ sequences. All 52 histologically proven tumors were visible.

Compared to the gold standard of the 2nd T1w SUB after contrast media application, the lesion diameter was comparable in SUB_Std_ and SUB_DL_ (mean SUB_Std_ 26.71, mean SUB_DL_ 26.63 for reader 1 and mean SUB_Std_ 30.89, SUB_DL_ 30.04 for reader 2; Table 5).

## 4. Discussion

Application of this new DL-based T1w VIBE in breast MRI at 1.5T led to superior image quality, sharpness, and SNR for both readers in all sequences with the same acquisition time of 73 s compared with the T1w VIBE_Std_ sequence. The DL technique reduces the noise, leading to a higher SNR, especially in lower-field strengths like 1.5T. In this study, DL-based T1w VIBE was employed to increase imaging quality, defined by higher resolution, while the scan time was kept unaltered to enable a comparable contrast media kinetics evaluation of the lesions. DL also enables a reduction in acquisition time while maintaining the SNR. However, this was not the case in this study as the contrast dynamics set the temporal scale.

As DL sequences provide denoised images, structures usually hidden by noise—like motion or aliasing artifacts—may become more pronounced [23]. In our study, comparable motion artefacts were detected for T1w VIBE_DL_ und T1w VIBE_Std_. However, our results reflect significantly more artefacts in T1w VIBE_DL_ pre- and post-contrast media application and the calculated SUB compared to the Std sequences. As the artefacts were detected in the mediastinum distant to the relevant images of the breast, diagnostic confidence was not affected.

As we performed T1w VIBE_DL_ sequences in addition to our clinical imaging standard, T1w VIBE_DL_ sequences were obtained at the end of the examination to provide our patients with the standard diagnostic image quality. Consequently, we compared the SUB of the last acquired T1w VIBE_Std_ with the SUB of T1 VIBE_DL_, in recognition of a higher number of motion artefacts in both sequences.

In our study, neither missed nor additional lesions in DL sequences pre-contrast and the diagnostically relevant SUB could be detected compared to standard sequences. In two patients, the tumor was not visible in the non-diagnostic unsubtracted T1w VIBE after contrast media application, affecting both the late Std and the DL T1w VIBE sequence without influencing the diagnostically relevant (early) SUB, however. Significantly higher detection rates of small liver lesions under 2 cm were reported in a study of DL controlled aliasing in parallel imaging results in higher acceleration (CAIPIRINHA) VIBE compared to the standard T1w VIBE in the hepatobiliary phase in 168 patients in the study by Wei et al. [24]. In this study, the DL sequences appeared to have a synthetic appearance.

### 4.1. Limitations

Limitations of this study encompass the relatively small study cohort of histologically proven breast cancer patients with different tumor entities. In the future, this sequence must be tested on larger patient cohorts, potentially with different histopathological subgroups. However, this study was planned as a feasibility study, providing the basis for additional studies in the future.

Furthermore, as mentioned in several other studies, we applied this new MR sequence to a private dataset at our institution at 1.5T. Broader examination using different scanners and field strengths should be performed in the future to prove the reproducibility [8,9].

Finally, we noticed a variation in the interrater reliability, limiting the objectivity of the visual analysis. Consequently, further studies should focus on the objective quantitative kinetic curve analysis. However, the effect of DL on dynamic contrast-enhanced MR analysis is still unclear [23].

### 4.2. Scientific Contribution

This study shows that this new DL T1w sequence in breast MR imaging at 1.5T provides superior image quality compared to conventional T1w sequences in clinical routine settings in a small prospectively examined study cohort of patients with histologically proven breast cancer.

The authors of a previous study investigated an accelerated high-resolution T1-weighted breast MR sequence with DL super-resolution reconstruction [25]. Analyzing 47 patients, they observed a higher overall image quality, SNR, and contrast-to-noise ratio (CNR) while reducing acquisition time by 51%. There was no loss in resolution due to super-resolution interpolation. The T1w VIBE_DL_ we used in our study uses the same acquisition time, compared to T1w VIBE_Std,_ and a gain in resolution is reached through super-resolution interpolation. An increased resolution is not only favorable in a collective of patients with biopsy-proven breast cancer but also in a high-risk screening collective. The aim of intensified surveillance in high-risk women, for example, is to find cancers at an early stage, and therefore, the detection of very small suspicious lesions is of great clinical importance.

The results of this study strengthen the role of DL in the future, that DL has not only the potential to decrease acquisition time but to also improve image quality without increasing acquisition time and therefore patients’ benefit from breast MR examination.

## 5. Conclusions

Deep learning-based T1w VIBE offers superior image quality in clinical routine at 1.5T compared to conventional T1w VIBE. Although T1w VIBE_DL_ was associated with significantly more artefacts, diagnostic confidence was not impaired, as the artefacts occurred in the mediastinum.

## Figures and Tables

**Table 1:** Overview of the protocol parameters of T1 VIBE_Std_ and T1 VIBE_DL_.

Protocol Parameter	T1 VIBE_Std_	T1 VIBE_DL_
Resolution	0.9 × 0.9 × 2.0 mm^3^	0.4 (i) × 0.4 (i) × 2.0 mm^3^
Matrix	448	544
FOV	420 × 420 mm^2^	420 × 420 mm^2^
TA/scan	73 s	73 s
Number of scans (Pre-/post-contrast)	1/7	1/1
TR/TE	7.73/4.77 ms	7.69/4.77 ms
Fat saturation	None	None
Parallel imaging factor	2	2
Partial Fourier (phase, slice)	7/8, 5/8	6/8, 6/8
Reconstruction mode	CAIPIRINHA	DL enhanced CAIPIRINHA with partial Fourier optimized super-resolution

**Table 2:** Results of the analysis of pre-contrast scans for T1 VIBE_Std_ and T1 VIBE_DL_. IQ = image quality; SNR = signal-to-noise ratio; DC = diagnostic confidence.

	Reader 1			Reader 2			Percentage of Agreement Between Raters	
Image Parameters T1 VIBE Sequence Precontrast	T1 VIBE_Std_ Mean (SD)	T1 VIBE_DL_ Mean (SD)	*p*-Value	T1 VIBE_Std_ Mean (SD)	T1 VIBE_DL_ Mean (SD)	*p*-Value	T1 VIBE_Std_	T1 VIBE_DL_
Motion artefacts	4.96 (0.19)	4.96 (0.02)	>0.99	4.98 (0.13)	4.96 (0.19)	0.322	94%	96%
Image quality (IQ)	4.17 (0.61)	4.91 (0.35)	<0.001	3.98 (0.13)	5.00 (0.00)	<0.001	60%	92%
Artefacts	4.96 (0.19)	5.000 (0.00)	0.159	4.75 (0.43)	4.30 (0.60)	<0.001	72%	38%
Sharpness	3.47 (0.54)	4.71 (0.57)	<0.001	3.04 (0.28)	4.98 (0.14)	<0.001	51%	76%
SNR	4.02 (0.58)	4.78 (0.57)	<0.001	4.00 (0.34)	4.92 (0.03)	<0.001	59%	84%
DC	4.51 (0.78)	4.67 (0.73)	0.004	4.39 (0.69)	4.61 (0.56)	0.001	61%	71%

**Table 3:** Results of the analysis of post-contrast scans for T1 VIBE_Std_ and T1 VIBE_DL_. * not available, as the difference between T1 VIBE_Std_ and T1 VIBE_DL_ for image quality is constantly calculated with 1. IQ = image quality; SNR = signal-to-noise ratio; DC = diagnostic confidence.

	Reader 1			Reader 2			Percentage of Agreement Between Raters	
Image Parameters T1 VIBE Sequence Postcontrast	T1 VIBE_Std_ Mean (SD)	T1 VIBE_DL_ Mean (SD)	*p*- Value	T1 VIBE_Std_ Mean (SD)	T1 VIBE_DL_ Mean (SD)	*p*- Value	T1 VIBE_Std_	T1 VIBE_DL_
Motion artefacts	4.94 (0.23)	4.89 (0.42)	0.261	4.98 (0.13)	4.96 (0.19)	0.322	92%	87%
Image quality (IQ)	4.04 (0.55)	4.75 (0.51)	<0.001	3.98 (0.13)	4.98 (0.13)	*	68%	77%
Artefacts	4.81 (0.39)	4.25 (0.55)	<0.001	4.75 (0.43)	4.30 (0.60)	<0.001	53%	70%
Sharpness	3.55 (0.57)	4.71 (0.64)	<0.001	3.06 (0.31)	4.92 (0.33)	<0.001	51%	76%
SNR	4.65 (0.60)	4.43 (0.74)	<0.001	4.06 (0.23)	4.92 (0.27)	<0.001	67%	75%
DC	4.43 (0.80)	4.53 (0.83)	0.058	4.59 (0.72)	4.82 (0.43)	0.001	63%	76%

**Table 4:** Results of the SUB for T1 VIBE_Std_ and T1 VIBE_DL_ (Note: The latest available post-contrast scan was always used for the subtraction). SUB = subtraction; IQ = image quality; SNR = signal-to-noise ratio; DC = diagnostic confidence.

	Reader 1			Reader 2			Percentage of Agreement Between Raters	
Image Parameters T1 VIBE SUB	T1 VIBE_Std_ Mean (SD)	T1 VIBE_DL_ Mean (SD)	*p*-Value	T1 VIBE_Std_ Mean (SD)	T1 VIBE_DL_ Mean (SD)	*p*-Value	T1 VIBE_Std_	T1 VIBE_DL_
Motion artefacts	4.25 (0.67)	4.09 (0.79)	0.10	4.02 (0.36)	4.00 (0.39)	0.766	55%	49%
Image quality (IQ)	3.79 (0.66)	4.58 (0.71)	<0.001	3.94 (0.30)	4.45 (0.63)	<0.001	70%	40%
Artefacts	4.83 (0.42)	4.36 (0.73)	<0.001	4.58 (0.49)	4.19 (0.55)	<0.001	47%	34%
Sharpness	3.57 (0.60)	4.83 (0.612)	<0.001	3.11 (0.32)	4.89 (0.37)	<0.001	43%	85%
SNR	4.17 (0.70)	4.83 (0.61)	<0.001	3.98 (0.23)	4.70 (0.54)	<0.001	53%	66%
DC	4.79 (0.68)	4.85 (0.63)	0.083	4.77 (0.46)	4.92 (0.26)	0.004	81%	91%

**Table 5:** Overview of the lesion size in reader 1 and 2.

Lesion Size in mm (Std)	Mean Value Reader 1	Mean Value Reader 2
SUB VIBE_Std_	26.71 (25.09)	30.89 (26.64)
SUB VIBE_DL_	26.63 (25.00)	30.04 (26.65)

## Data Availability

The data presented in this study are available from the corresponding author upon request due to legal reasons.

## Abbreviations

The following abbreviations are used in this manuscript:

BI-RADS	Breast Imaging Reporting and Data System
BRCA	Breast cancer gene
CAIPIRINHA	Controlled aliasing in parallel imaging results in higher acceleration
CNR	Contrast-to-noise ratio
DL	Deep learning
DCIS	Ductal carcinoma in situ
DCE MRI	Dynamic contrast-enhanced MRI
EUSOBI	European Society of Breast Imaging
ILC	Invasive lobular breast carcinoma
NST	No special type
ROI	Region of interest
SNR	Signal–to-noise ratio
SUB	Subtraction
TA	Acquisition time
TE	Echo time
TR	Repetition time
VIBE	Volumetric interpolated breath-hold
W	Weighted
WIP	Work-in-progress
